# The First Complete Genome Sequence of a Novel *Tetrastichus brontispae* RNA Virus-1 (TbRV-1)

**DOI:** 10.3390/v11030257

**Published:** 2019-03-13

**Authors:** E Meng, Baozhen Tang, Francisco Javier Sanchez-Garcia, Ting Qiao, Lang Fu, Yu Wang, You-Ming Hou, Jiang-Lin Wu, Zhi-Ming Chen

**Affiliations:** 1 State Key Laboratory of Ecological Pest Control of Fujian-Taiwan Crops, Fujian Agriculture and Forestry University, Fuzhou 350002, China; menge1987@126.com (E.M.); tbyun@126.com (B.T.); 000b182737@fafu.edu.cn (F.J.S.-G.); qiaoting1209@126.com (T.Q.); m18305918768@163.com (L.F.) 18006907901@163.com (Y.W.); jianglinwu99@gmail.com (J.-L.W.); 2 Qingzhou Yatai Agricultural Technology Company Limited, Qingzhou 262500, China; 3 Fujian Provincial Key Laboratory of Insect Ecology, College of Plant Protection, Fujian Agriculture and Forestry University, Fuzhou 350002, China; 4 Fuzhou Entry-Exit Inspection & Quarantine Bureau of P.R.C, Fuzhou 350002, China; czmfzciq@163.com

**Keywords:** dimarhabdovirus, negative-sense (–) single-stranded RNA virus, viral genome, insect virus, phylogenetic analysis

## Abstract

The complete sequence of a novel RNA virus isolated from *Tetrastichus brontispae* (TbRV-1) was determined to be 12,239 nucleotides in length with five non-overlapping, linearly arranged coding sequences (CDS), potentially encoding nucleoproteins, hypothetical proteins, matrix proteins, glycoproteins, and RNA-dependent RNA polymerases. Sequence analysis indicated that the RNA-dependent RNA polymerase of TbRV-1 shares a 65% nucleotide and 67% amino acid sequence identity with Hubei dimarhabdovirus 2, suggesting that TbRV-1 is a member of the dimarhabdovirus supergroup. This corresponded to the result of the phylogenetic analysis. The affiliation of TbRV-1 with members of the family Rhabdoviridae was further validated by similar transcription termination motifs (GGAACUUUUUUU) to the *Drosophila* sigmavirus. The prevalence of TbRV-1 in all tissues suggested that the virus was constitutive of, and not specific to, any wasp tissue. To our knowledge, this is the first report on the complete genome sequence of a dimarhabdovirus in parasitoids.

## 1. Introduction

Rhabdoviridae, a family of viruses in the Mononegavirales order, are negative-sense (–) single-stranded RNA viruses. Most of them infect plants and animals (mammals, birds, reptiles, fish and arthropods) and are categorized into arthropod-vectored viruses of plants or vertebrates, and vertebrate- and arthropod-specific viruses on the basis of the host range [1]. Based on the strong nexus of virus clustering and the host types, as well as the broad host range of some rhabdovirus, the insects are proposed to be the primary determinants of host range of the plant and animal rhabdovirus [2,3]. Currently, 20 officially recognized genera and one viruses are reported [4]. The main insect-associated rhabdoviridae are from the genera *Vesiculovirus*, *Cytorhabdovirus*, *Nucleorhabdovirus*, *Sigmavirus*, *Ephemerovirus* and the superfamily dimarhabdovirus [1], where the majority insect vectors are from Diptera, Hemiptera, and Lepidopteara [3].

Among the arthropod-specific viruses, one group of predominantly insect-associated viruses is a sister group to the large clade of plant viruses, while the other group is the sigma virus clade, and are derived from the vector-borne dimarhabdoviruses [3]. Dimarhabdovirus, the dominant type of vector-borne rhabdovirus, which mainly infects Diptera, is the causative agent of significant morbidity and mortality among humans and animals globally [5]. In contrast to the cytolytic sides of rhabdoviruses to the mammalian, little or no cell killing is observed when insects are infected [6]. The rhabdoviral infection of an insect is typically persistent, and includes an initial phase of rapid replication, an acute phase of viral production, and a persistent phase with lower viral replication levels [6]. Even so, most insects infected with rhabdoviruses become more sensitive to paralysis and even die on exposure to CO_2_, such as the effect of sigmavirus on *Drosophila* [7,8]. Up to now, most research on insect-associated rhabdovirus still focuses on the initial characterization, with fewer reports on their roles.

Parasitoid wasps as vital biocontrol agents against pests are widely used in agro-ecosystems. In most cases, the higher efficiency of the parasitism ratio of Icheneumonidae and Braconidae wasps results in the presentation of viruses or virus-like particles detected in parasitoid wasps, such as the polydnavirus [9,10,11,12]. In other cases, the viruses presented in parasitoid wasps affect reproductive efficiency. For example, the *Pteromalus puparum* negative-strand RNA virus 1 (PpNSRV-1) reduced female offspring numbers, while it increased the longevity of the wasp *P. puparum* [13]. For the rhabdovirus from Hymenoptera, the *Diachasminorpha longicaudata* rhabdovirus (DlRhV) was introduced into the host *Anastrepha suspense* upon parasitism, and no indispensable role of DlRhV in successful parasitism was reported [14,15]. In addition, the genome of DlRhV was sequenced in 2016, and still is the only known genome of rhabodovirus from Hymenoptera [15].

*Tetrastichus brontispae*, a type of endoparasitoid wasp, is an efficient bio-control agent against several invasive leaf beetle species, such as *Octodonta nipae* and *Brontispa longissima* [5,16,17,18,19]. Previous studies showed that a novel mixture of systemic active and local active regulation was used for *T. brontispae* to escape host encapsulation in *O. nipae* pupae [20]. The role of virus-like particles and calyx fluid in achieving local active regulation has been widely reported [21,22]. Hence, the types of virus present in *T. brontispae* were detected by next generation sequencing (NGS).

In this paper, the detection of virus types was performed by metatranscriptional analysis, and the complete genome of a novel dimarhabdovirus from *T. brontispae*, named *T. brontispae* RNA virus 1 (TbRV-1) was reported. The TbRV-1 was somewhat similar to Hubei dimarhabdovirus, a novel virus discovered in Odonata by transcriptome and assigned to the dimarhabdovirus superfamily by bioinformatics [23].This study broadens the current knowledge on the fundamental patterns and processes of the viral evolution of rhabdoviruses.

## 2. Materials and Methods

### 2.1. Insect Collection

The wasps, *T. brontispae*, were reared on *Octodonta nipae* or *Brontispa longissima* (Coleoptera: Chrysomelidae) on the campus of Fujian Agriculture and Forestry University for nearly four years, after being collected from the Chinese Academy of Tropical Agricultural Sciences in 2012. 

### 2.2. Transcriptome of the Tetrastichus brontispae Abdomen

The transcriptome of *T. brontispae* was sequenced as described by Tang et al. (2014) [24]. The total RNA from the 575 abdomens of female wasps was extracted using the Trizol reagent (Invitrogen, New York, NY, USA) and treated with DNase I. The concentration and integrity of the total RNA were assessed by a 2100 Bioanalyzer (Agilent Technologies, Santa Clara, CA, USA) before library construction. 

The complementary DNA (cDNA) library was constructed with NEBNext® Ultra RNA Library Prep Kit (New England Biolabs, Ipswich, MA, USA). Poly (A) mRNA was isolated using oligo-dT beads (Qiagen, Tokyo, Japan). All mRNA was broken into short fragments (200 nt) by adding a fragmentation buffer. First-strand cDNA was generated using random hexamer-primed reverse transcription, followed by the synthesis of the second-strand cDNA using RNase H and DNA polymerase I. The cDNA fragments were purified using a QIAquick PCR extraction kit. These purified fragments were then washed with EB buffer for the addition of end reparation poly (A), and then they were ligated to sequencing adapters. Following agarose gel electrophoresis and the extraction of cDNA from gels, the cDNA fragments (200 ± 25 bp) were purified and enriched by PCR to construct the final cDNA library. The cDNA library was qualified and quantified with an Agilent 2100 Bioanalyzer and an ABI StepOnePlus Real-time PCR system, respectively, and then sequenced for 125 bp on the Illumina HiSeq ^TM^ 2500 platform using the single-end/paired-end technology in a single run at Genedenovo (Guangzhou, China).

### 2.3. Viral Metagenomics Analysis

For the reads derived from RNA-Seq, the clean data was obtained by filtering out unknown nucleotides larger than 5% and low quality reads and used for assembly with Trinity [25]. Those assembled sequences were outputted as unigenes. Unigenes aligned to Rhabdoviridae by Blastx with an *E*-value cut-off of 1 × 10^−5^ against the NCBI non-redundant (NR) database were chosen for further analysis because of the scarce reports regarding the Rhabdoviridae in Hymenoptera. 

### 2.4. Virus Genome Sequencing

The canonical genome structure of rhabdovirus is 3’-nucleoprotein (N)-phosphoprotein (P)-matrix protein (M)-glycoprotein (G)-RNA-dependent RNA polymerase (L) [26]. Based on the typical genome layout of rhabdovirus (3’-N-P-M-G-L-5’), the gaps among the unigenes of TbRV-1 were first amplified by a routine polymerase chain reaction. And the gaps between the genes and the terminus end of TbRV-1 were obtained by the rapid amplification of cDNA ends (RACE PCR) with a SMARTer^TM^ RACE PCR kit (Clonetech, Shiga, Japan). The specific primers for amplifying the whole genome of the putative rhabdovirus were designed according to the obtained sequences (Supplementary Table S1).

The PCR products were sequenced by Invitrogen (Guangzhou, China) after ligation into the T1 vector (TransGen Biotech, Beijing, China) and transformation into *Escherichia coli* T1. The final sequences were assembled using the DNAMAN 7 program (Lynnon, Quebec, Canada). The full length of the coding sequence was further validated after sequencing the products of the routine PCR acquired with a fastfly pfu enzyme (TransGen Biotech).

The complete genome of TbRV-1 was aligned to the known viruses by Blastx and Blastn to identify its closest relatives. The putative open reading frames (ORFs) were predicted by the ORF finder (https://www.ncbi.nlm.nih.gov/orffinder/). ORFs were analyzed on InterPro Scan [27] and PROSITE databases to find matches to known protein families and the potential functions of predicted proteins [28]. A maximum likelihood phylogenetic tree constructed with RdRP, the most conservative sequence domain across all RNA viruses [23,26], and was performed by MEGA 5.2 using the rtREV substitution model with gamma distributed and invariant sites (G + I) and the bootstrap method with 1000 repetitions [29]. The information on the sequences used for the analysis is listed in Supplementary Table S2. 

### 2.5. MicroRNA Library Construction and Sequencing

As suggested earlier, infection caused by arboviruses is typically persistent, and is characterized by an initial phase of rapid replication and substantial viral production [6]. In response to infection, invertebrates process replicating viral RNA genomes into short interfering RNAs (siRNAs) of discrete sizes to guide virus clearance by RNA interference [30]. Therefore, to detect if the TbRV-1 were replicating in the wasps, the profile of small RNA tags from TbRV-1 was searched.

The small RNA library was constructed as follows. Seventy individuals of *T. brontispae* were used for every library. The total RNA was extracted by Trizol reagent. The RNA molecules in a size of 18–30 nts (nucleotides) were enriched from the total RNA of *T. brontispae* by polyacrylamide gel electrophoresis. After the ligation of 3’ and 5’ adapters, the ligation products were reverse-transcribed by PCR amplification, where the PCR products were enriched to generate a cDNA library and sequenced using IlluminaHiseq^TM^ 2500 by Gene Denovo (Guangzhou, China).

Before further analysis, the clean tags were retrieved by trimming the adaptors after removing low quality reads containing more than one low quality base (*Q*-value ≤ 20) or unknown nucleotides. The clean reads were de novo assembled by Velvet with a k-mer of 17 [31]. To check if there were small RNA tags from TbRV-1, the assembled reads were subjected to Blastn analysis against the genome of TbRV-1 with a threshold *E* value of 10^−5^.

### 2.6. Isolation of Virus RNA

The virus pellet was isolated following the procedure described by Suzuki and Tanake (2005) [32], with some modification. Briefly, the wasps *T. brontispae* were homogenized with pipette. Then, the supernatants drawn from the homogenization after centrifuging at 600 g for 10 min were used for re-centrifuging at 15,000 g for 30 min at 4 °C. The virus pellets were resuspended in sterilized PBS, filtered with 0.45 μm Millipore filter, and used for RNA extraction with Qiagen virus RNA extraction kit (Qiagen, Tokyo, Japan). Then, the RNA was visualized by 1% agarose gel electrophoresis.

To detect if there was TbRV-1 in the mixture, the RNA extracted above was reverse-transcribed to cDNA using the SMARTer^TM^ RACE cDNA amplification kit (Clontech laboratories Incorporation, Japan). A pair of primers (MS and MA), amplifying 1541 bp, was used for PCR amplification. The PCR was run with fastfly pfu enzyme with the following conditions: 30 s at 94 °C, 30 s at 49.8 °C, and 45 s at 72 °C for 35 cycles. The PCR reaction, with distilled water as the template, was used as a negative control. The PCR results were visualized by agarose gel electrophoresis.

### 2.7. Tissue Distribution and Developmental Expression Patterns of TbRV-1

The main parasitism factors of endoparasitoids are located in the ovaries and venom apparatus, while the gut appears to be the main barrier that must be circumvented for transmission [2,33]. Therefore, the tissue tropism of TbRV-1 was measured to obtain a better conjecture of the role of TbRV-1. The total RNA copy numbers of TbRV-1 in the head, thorax, venom apparatus, ovaries, and gut from the female wasp and the whole male body were measured. The gene used for the quantification was *RdRP*, which codes the protein required for transcription, plays a role in genome replication, and is the most conserved sequence domain among RNA viruses [23].

For quantification, the total RNA (about 800 ng RNA, 500 wasps) extracted from the above tissues was subjected to the Thermo Scientific Verso cDNA kit (Thermo Fisher Scientific Incorporation, Waltham, MA, USA) to synthesize the cDNA as a template, where the RT enhancers were used to remove the contaminating genomic DNA. The abundance of RdRP gene copies (copies/μL) among tissues was measured with real time quantitative PCR (qPCR) using ABI 7500 after constructing the absolute standard curve. Before qPCR, a recombinant plasmid named pEASY-Blunt-RdRP was constructed by inserting the purified PCR product of RdRP to the pEASY-Blunt cloning vector (TransGen Biotech, Beijing, China). Then, the recombinant plasmid pEASY-Blunt-RdRP was digested with the endonuclease Sal I (New England BioLabs, USA). The digested plasmid was diluted to 10^2^, 10^4^, 10^5^, 10^6^, and 10^7^ with distilled water for the absolute standard curve construction. Three biological and two technical repeats were performed. The primers for this study are shown in Supplementary Table S1. The non-template control was included in every plate. The amount of TbRV-1 was compared using a one-way ANOVA analysis, followed by Tukey’s multiple comparison test (*p* < 0.05).

## 3. Results

### 3.1. Five Types of RNA Virus Present in the T. brontispae Transcriptome

The Tetrastichus brontispae RNA virus 1 (TbRV-1), Tetrastichus brontispae RNA virus 2 (TbRV-2), Tetrastichus brontispae RNA virus 3 (TbRV-3), Tetrastichus brontispae RNA virus 4 (TbRV-4), and Tetrastichus brontispae RNA virus 5 (TbRV-5) were identified through transcriptome mining (Table 1). The Blast results showed that TbRV-1, TbRV-2, TbRV-3, TbRV-4, and TbRV-5 were aligned to the viruses that belonged to Rhabdoviridae, Orthomyxoviridae, Iflaviridae, Narnaviridae, and Reoviridae families, respectively. Considering the rare reports of rhabdovirus in Hymenoptera, the genome and organization of TbRV-1 are further dicussed.

### 3.2. The Genome Organization of TbRV-1

A sequence of 12,239 nts (nucleotides) flanking 98 nt from the 5’ leader and 112 nt from the 3’ trailer was assembled for TbRV-1 (GenBank accession number: MH643740), where A+T pairs comprised 69.5% of the nucleotides. By comparing the 5’/3’untranslated regions (5’ UTR and 3’ UTR) and intergenic regions of the TbRV-1 genomes, the putative transcription termination motifs (TTP: GGAACUUUUUUU) similar to the TTP motif G(U)_7_ of the *Sigmavirus* from *Drosophila* [34,35] and the different putative conserved transcription initiations (Ti: GAUUKRU) of the *Sigmavirus* (Ti, GUUGUNG ) were observed. These conserved Ti and TTP typically flanked the structural genes and made the genes independently transcribed [34,36].

**Figure 1:**
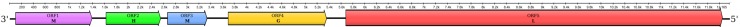
Schematic representation of the genome organization and location of each putative open reading frame (ORF). N: nucleoprotein; H: hypothetical protein; M: matrix protein; G: glycoprotein; and L: RNA-dependent RNA polymerase.

TbRV-1 has five putative ORFs (ORF I–V), located at nt positions 113–1393 (N), 1689–2600 (H), 2699–3355 (M), 3761–5410 (G), and 5781-12141 (L). These five ORFs, except for ORF II, have a conserved domain and are aligned to the corresponding proteins of Hubei dimarhabdovirus 2 or the Wuhan insect virus 7 by Blastp, rather than Blastn (Table 2). According to the bioinformatics analysis, the TbRV-1 putatively encoded N, H, M, G, and L (Figure 1), which is somewhat analogous to the general organization of the rhabdovirus genome structure “N-P-M-G-L” [25]. The P of the rhabdovirus is a multifunctional protein that plays multiple roles in viral transcription and replication processes [37], although it is also coupled with a high rate of evolution. The highly divergent protein sequence of phosphoprotein among rhabdoviruses, shown as Supplementary Figure S1, may be related to the highly selective pressures of its hosts, such as the RNAi machinery [38], which needs further validation. 

### 3.3. Detection of TbRV-1 siRNAs

After a thorough Blastn analysis against the genome of TbRV-1, three corresponding small RNA sequences were detected. The corresponding coverages of these three sequences were 3122–3163, 7790–7831, and 11037–12078, with 100% identity. In detail, the first two sequences were aligned to ORF III (putative Matrix protein), and the last two sequences were aligned to ORF V (putative RdRP Protein). The sequence of the three unigenes and the blast results are shown below (Table 3).

### 3.4. Identification of Virus RNA from the Isolated Mixtures

**Figure 2:**
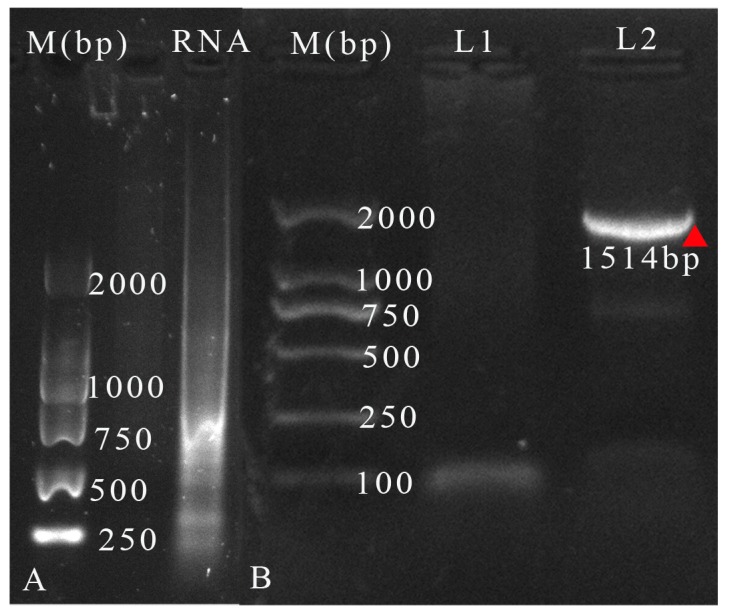
(**A**) Gel electrophoresis of viral RNA, and (**B**) the PCR products obtained from the viral cDNA and distilled water. M: Marker DL 2000 (TransGen Biotech); L1: PCR products with distilled water as template; L2: PCR products with cDNA template. The band was marked with a red triangle.

After filtration, RNA extraction with the Qiagen kit was mainly a mixture of virus RNA. After agarose gel electrophoresis, a smear was observed in the respective lane when the extracted RNA was loaded into the gel (Figure 2A). For PCR validation, only the PCR with the cDNA as a template showed a visible band, while the products of negative PCR did not (Figure 2B). This further validated the presence of TbRV-1 in the mixture.

### 3.5. The Quantification of Virus RNA Copy Numbers

The standard curves of RdRp were calculated using the number of the copies and the corresponding cycle threshold (ct). The equation is *y* = −0.282*x* + 10.03, *R*^2^ = 0.9994, where *y* is the number after the log function of copies/ng of the genes, and *x* represents the corresponding *ct*. The gene copies of TbRV-1 were the two-folded reduction of the calculated values, as double-stranded plasmid was used for standard curves. The amplification efficiency in this range was 91.02%, the respective limitations of quantification of the RdRP gene copies were 18.9–1.89 × 10^6^, and their corresponding *ct* values were 13.43–31.00. The quantitative analysis did not detect any significant differences in the gene copies of RdRP among the tissues (F_4,10_ = 1.706, *p* = 0.225) (Table 4).

### 3.6. Phylogenetic Analysis Using the Protein Sequence of RdRP

**Figure 3:**
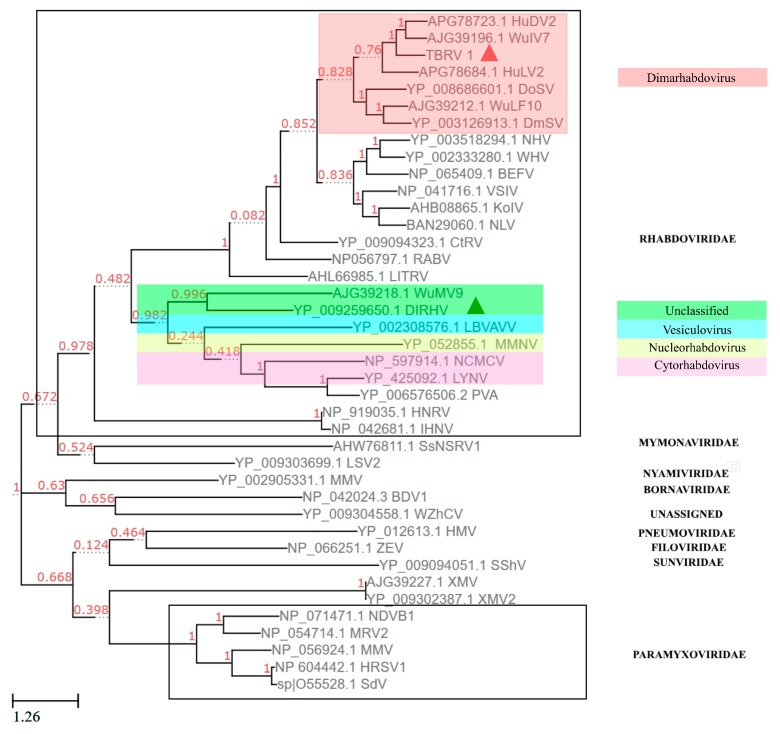
Maximum likelihood phylogenetic tree of TbRV-1 based on the RdRP protein and alignment. The numbers near the branches represent the percentage of the 1000 bootstrap iterations supporting the branches. The phylogeny was drawn with ETE Toolkit v3 software. The virus TbRV-1 and DlRhV were respectively marked with red and green triangles.

The phylogenetic analysis-based RdRP showed that the TbRV-1 was clustered into a clade with two dimarhabdoviruses: Hubei dimarhabdovirus 2 (HuDV2, Accession number: KX884426) and Wuhan insect virus 7 (WuIV7, Accession number: KM817653.1) at high bootstrap values (BP = 100%, Figure 3), where both dimarhabdoviruses were newly discovered by RNA-seq sequencing [23,39]. The DlRhV was clustered with other unassigned viruses, and was more familiar with vesiculovirus. With a lower similarity (25% identity, *e* = 1 × 10^−81^) in the amino acid sequence of RdRp to DlRhV, another rhabdovirus from parasitoids TbRV-1 was observed. In all, TbRV-1 is a novel virus, different from the previously reported DlRhV.

## 4. Discussion

The genome of TbRV-1 exhibited a significant divergence from DlRhV by Blastn and Blastp, but a higher similarity to two dimarhabdovirus supergroups, HuDV2 and WuIV7 [23,39]. Other supports for this finding are the similarly conserved TTP domains and the clusters with the *Sigmavirus* from *Drosophila*, which is a member of the dimarhabdovirus group [40], while the DlRhV, another rhabdovirus isolated from parasitoid, is more closely related to other arthropod-infecting rhabdoviruses, but distantly related to the sigma viruses [15]. This is the first report of a dimarhabdovirus in an endoparasitoid, where the general insect host of dimarhabdoviruses are dipteran insects, such as *Ochlerotatus* sp., mosquitoes [26,41], *Drosophila* [41], and *Odonata* [23].

To fight against virus infection, the replicating viral RNA genomes were processed into small interfering RNA (siRNAs) by invertebrates [30]. In accordance with this, three siRNAs were retrieved from a small amount of RNA data. The low frequencies of siRNA in the small RNA library may also indicate a lower infection rate of this virus. Further, lower RNA copy numbers were quantified in the detected tissues, which imply that the virus might have a long latent period that is required for the rhabdovirus to exist in a vector insect before transmission [2,42]. The presence of TbRV-1 in all tissues of female and male wasps suggests that the expression of TbRV-1 is constitutive of, and not specific to, any wasp tissue. 

The evolutionary challenge faced by RNA viruses is to maximize functional diversity with several constraints concerning the genome size. For dimarhabdoviruses, the evolution of genomic plasticity has been associated with that of alternative, overlapping and consecutive ORFs within the major structural protein genes as well as the insertion and loss of additional ORFs in each gene junction in a clade-specific manner [34]. In this study, the differences in the genome structure of TbRV-1 and the *Sigmavirus* clade are observed where the genome of TbRV-1 lacked a sigmaX gene, which is characteristic of *Sigmavirus*. Nonetheless, it cannot be inferred from this that TbRV-1 is classified as a novel species or genus because the sequence comparisons alone cannot be the only source in developing the classification schemes of viruses at this time, [43]. Meanwhile, the ability to accurately read the sequence information and robustly infer the phenotypic data is also fundamental for virus taxonomy based on the sequence information alone [44].

In general, viruses or virus-like particles (VLPs) in parasitoid wasps (Hymenoptera: Apocrita), such as the well-known polydnaviruses [9,10,45], VLPs [46,47,48,49], and some other viruses such as reoviruses, facilitate their parasitism success [50]. For example, the iflavirus, named *Dinocampus coccinellae* paralysis virus (DcPV), can induce changes in the lady beetle (*Coleomegilla maculate*), such as tremors, gait disturbance, and limitations in movement during parasitism [51]. The *P. puparum* negative-strand RNA virus 1 (PpNSRV-1), a novel member of *Nyamiviridae*, impaired several fitness parameters of the wasp, such as adult longevity and the offspring sex ratio but also affected parasitism [13]. For rhabdoviruses, only one virus, DlRhV, was observed in the venom apparatus of *D. longicaudata* parasitoids, and no role of DlRhV in parasitism was ascertained [14,15]. In this study, the TbRV-1 viruses were also transcribed in the venom apparatus, a major organ filled with parasitoid factors, which may be an indicator of the potential roles of TbRV-1 in parasitism. In addition, Longdon et al. (2010) discussed the sensitivity of the host, such as *Drosophila* or the mosquito, with rhabdovirus to CO_2_ and its negative effects, such as the reduced longevity [41]. The experiments on the role of TbRV-1 on anoxia, and the potential role on parasitism are underway. Meanwhile, for a more detailed study on the role of TbRV-1, we are establishing a virus cell culture system.

## Figures and Tables

**Table 1:** Virus species identified through transcriptome mining.

Query Virus	Gene ID	Length (bp)	Subject Protein	Subject Virus	*E* Value	Taxonomy of Subject Virus	Coverage Range
*Tetrastichus brontispae* RNA virus-1 (TbRV-1)	Unigene0017563	1431	Nucleoprotein	Oita rhabdovirus 296/1972	8.2 × 10^−100^	Rhabdoviridae (negative sense single strand RNA viruses)	1–1431
	Unigene0019568	1954	Matrix protein	Tibtogaran virus	9 × 10^−6^		1442–3395
	Unigene0023051	5167	RdRp	Scophthalmus maximus Rhabdovirus	0		3411–8578
	Unigene0014453	1037	RdRp		2 × 10^−21^		11,075–12,171
	Unigene0016900	339	RdRp		4 × 10^−35^		9036–9375
	Unigene0016901	404	RdRp		4 × 10^−33^		8656–9058
	Unigene0008666	895	RdRp		1 × 10^−35^		9744–10,603
	Unigene0005445	216	RdRp	Grass carp rhabdovirus V76	9 × 10^−13^		10,581–10,796
*Tetrastichus brontispae* RNA virus -2 (TbRV-2)	Unigene0000118	2396	Polymerase PB1	Quaranfil virus	0	Orthomyxoviridae (negative sense single strand RNA viruses, 6–8 segments)	/
	Unigene0022762	2405	Polymerase PB1		7 × 10^−171^		
	Unigeno0022160	1447	Polymerase PA		1 × 10^−36^		
	Unigene0018460	2416	Polymerase PA		6 × 10^−39^		
	Unigene0020769	2500	Polymerase PB2		2 × 10^−21^		
	Unigene0000506	427	Polymerase PB2		2 × 10^−7^		
	Unigene0021353	1762	Hypothetical protein		1 × 10^−36^		
	Unigene0021482	1772	Hypothetical protein		4 × 10^−20^		
	Unigene0016137	1656	Hemagglutinin		2 × 10^−32^		
	Unigene0020778	1614	Hemagglutinin		3 × 10^−42^		
*Tetrastichus brontispae* RNA virus 3 (TbRV-3)	Unigene000903	7027	Polyprotein	Deformed wing virus	1 × 10^−69^	Iflaviridae	/
	Unigene000905	2933	Polyprotein		4 × 10^−101^		
*Tetrastichus brontispae* RNA virus -4 (TbRV-4)	Unigene0022228	2784	RdRp	Saccharomyces 23S RNA Narnavirus	5 × 10^−18^	Narnaviridae (positive sense single strand RNA viruses)	*/*
*Tetrastichus brontispae* RNA virus -5 (TbRV-5)	Unigene0008109	260	VP3	Kadipiro virus	5 × 10^−8^	Reoviridae (double strand RNA viruses)	*/*
	Unigene0002126	2047	VP4		3 × 10^−109^		
	Unigene0011479	1396	VP5		4 × 10^−46^		
	Unigene0004982	1092	VP7		2 × 10^−27^		
	Unigene0005372	850	VP12		3 × 10^−22^		
	Unigene0012923	483	RdRp	Liaoning virus	1 × 10^−26^		
	Unigene0012924	204	RdRp		1 × 10^−12^		
	Unigene0013487	253	VP1		4 × 10^−18^		
	Unigene0002892	281	VP3		9 × 10^−21^		
	Unigene0010175	228	VP2		1 × 10^−17^		
	Unigene0016608	1775	VP2		2 × 10^−88^		
	Unigene0016692	426	VP2		1 × 10^−27^		
	Unigene0016693	270	VP2		1 × 10^−16^		
	Unigene0006368	206	VP3		2 × 10^−13^		
	Unigene0005945	251	VP3		3 × 10^−17^		
	Unigene0011375	856	VP6		1 × 10^−26^		
	Unigene0008523	1035	VP8		8 × 10^−48^		
	Unigene0014543	278	RdRp		3 × 10^−14^		
	Unigene0006593	250	RdRp		4 × 10^−16^		
	Unigene0007896	425	Capping enzyme		1 × 10^–13^		
	Unigene0001578	236	VP1	Banna virus	2 × 10^–12^		

**Table 2:** Calculated and predicted properties of TbRV-1 (*Tetrastichus brontispae RNA virus 1)* ORFs.

Virus ORF	Position (bp)	ORF Length (nt)	Protein Length	Protein Mass (kDa)	pI	Signal Peptide	No. of O-linked Glycosylation Sites	No. of Phosphorylation Sites			Top Blastx Match	*E* Value	Domain	*E* Value of Domain
								T	Y	S				
ORF I	113–1393	1287	426	49.27	6.92	NO	9	16	9	26	Putative nucleoprotein (Hubei dimarhabdovirus 2)	1 × 10^−100^	Rhabdo-ncap	2.78 × 10^−64^
ORF II	1689–2600	912	303	34.79	5.50	NO	13	11	3	15	ND	-	ND	-
ORF III	2699–3355	657	218	25.97	6.91	NO	0	10	6	7	Matrix protein (Hubei dimarhabdovirus 2)	2 × 10^−22^	Vesiculo_matrix super family	2.49 × 10^−4^
ORF IV	3761–5410	1650	549	63.28	7.45	NO	2	19	9	27	Glycoprotein (Wuhan insect virus 7)	2 × 10^−61^	Rhabdo_glycop super family	1.78 × 10^−23^
ORF V	5781–12,141	6360	2119	245.17	8.27	NO	10	67	38	113	RdRp (Hubei dimarhabdovirus 2)	0	Mononeg_RNA-pol super family; Methyltrans_Mon super family; Mononeg_mRNAcap super family;	01.15 × 10^−132^; 4.61 × 10^−50^

ND means no significant homology was detected.

**Table 3:** The Blastn results of small interfering RNA against the genome of TbRV-1.

Library	Small Interfering RNA Sequence	Identity	Coverage Range (nt)	Domain
TbBL1	CAGGTTTTTTATTATTTTATCCTCTTGATTTGTGTCTCTAAC	100%	11,037–11,078	RdRP
TbBL2	TTGGAGTGTTGTTAGTGCATTAGCCATAGAAAGGGAATCCAA	100%	7790–7831	RdRP
TbBL3	TTTAAGTTTAAATAAAATATTAACCTGATGATCTGGAGCTCC	100%	3122–3163	Matrix

Note: TbBL1, TbBL2, and TbBL3 represent three small RNA libraries of the *Tetrastichus brontispae* reared on *Brontispa longissima*. The column of domain shows in which domains the three separate siRNA were blasted. RdRP: RNA-dependent RNA polymerase; Matrix: putative matrix protein.

**Table 4:** Viral loads of TbRV-1 in different tissues of *T. brontispae* wasps.

Sex	Tissues	Copies/ng Total RNA
Female wasps	Head and thorax	690 ± 189 a
	Ovary	706 ± 120 a
	Venom apparatus	446 ± 130 a
	Remaining abdomen	376 ± 6 a
Male wasps	Whole body	376 ± 119 a

Data represent means ± standard error. The columns annotated with the same letters are not significantly different (Tukey’s multiple comparison tests). The copies were obtained by a two-fold reduction of the calculated value.

## Supplementary Materials

The following are available online at https://www.mdpi.com/1999-4915/11/3/257/s1, Figure S1: Amino acid sequences alignment of the phosphoprotein of rhabdovirus. Similar residues are boxed and marked red, Table S1: Primers used in this study, Table S2: Sequences used for phylogenetic tree construction in this study.
