# Supplementary material for: The First Complete Genome Sequence of a Novel Tetrastichus brontispae RNA Virus-1 (TbRV-1)
Source: Viruses. 2019 Mar 13;11(3):257. doi: 10.3390/v11030257 (PMC6466307; doi:10.3390/v11030257)
Supplement: Supplementary file 1 [file viruses-11-00257-s001.zip › supplementary files/Supplementary Figure S1.pdf]

```
                                          1        10                  20        30        40
ASZ85160.1_phosphoprotein_Rhabdoviridae   MDLEGNEEIR....AL...............MAQYPELPTALDEVAAISREV.DAEDDYD.....
sp|Q9E7N7|_Phosphoprotein_Lettuce_necro   MDSESLDFSSAD.TVI...............LRSPN....AGTNP..............D.....
sp|P06747|PHOSP_RABVP_Phosphoprotein      MSKI.FVNPS....AI...............RAGLADLEMAEETVDLINRNI.EDNQAH......
sp|Q9E787|PHOSP_BEFVB_Phosphoprotein      ....................MSHLKPKIMTGAYDAEKLRRNLQEQIALEEDELENQTDFESNKEE
sp|Q82681|PHOSP_IHNVW_Phosphoprotein      ...................MSDGEG.EQFF...........NLEGE....DILRLESRLKTPRND
sp|Q6E0X0|PHOSP_MMVR_Phosphoprotein_OS_   MNRYSRRSRHP..NPP..................VP....NQEEP..............E.....
sp|P03520|PHOSP_VSIVA_Phosphoprotein_OS   ..MDNLTKVR....EY...............LKSYSRLDQAVGEIDEIEAQR.AEKSNYE.....
gi|1070619313|ref|YP_009301740.1|_putat   MKQQNYIYPSMELRNL.TGPKKFFASGKGYLTKEYRNS..PFAEQGQLAVATNPRRTSWAMSDEE
TbRV_1_unnamed_protein_product            MNPKHPLLNPKL.TRLINNKRVNVNSMINCFESTLPETIDNLNEIDQIQMQKTPGN..W..DLQD

                                                                    50         60         70
ASZ85160.1_phosphoprotein_Rhabdoviridae   ..LP..............S..DA..PRIDFDSDSEDEDA.....DE......ADLDRPVSF....
sp|Q9E7N7|_Phosphoprotein_Lettuce_necro   ....G..........HP.D..TV..ECPDFDTDIPKTSD.....DS......SKMDNKGSSSSSK
sp|P06747|PHOSP_RABVP_Phosphoprotein      ..LQG.............E..PI..EVDNLPEDMGRLHL.....DD......GKSPNPGEM....
sp|Q9E787|PHOSP_BEFVB_Phosphoprotein      SNINNNP.LVIKQ..EP.SIY....PIEINL.EDLEKAN.....GMEEDWENSL..MKIIESSDV
sp|Q82681|PHOSP_IHNVW_Phosphoprotein      GQIGKNP.RRRKEDQAP.........QEEPKKTTRRP................D..KNKGLSQLE
sp|Q6E0X0|PHOSP_MMVR_Phosphoprotein_OS_   ....R..........DP.N..HI..............DQ.....DL......ADLAQPLVLK...
sp|P03520|PHOSP_VSIVA_Phosphoprotein_OS   ..LFQ.........E.D.G..VE..EHT.KPSYFQAADDSDTESEP......EIEDNQGLYAQD.
gi|1070619313|ref|YP_009301740.1|_putat   GNIKD..LG....DAFQKE..LEQKHSSDSDSQTSVSSN.....DT......VKMDPPP...PPP
TbRV_1_unnamed_protein_product            SDIESDDDCILSQ..KG.KDFQL..ECDMFD...PKSVI.....QS......THITNENHNDKPG

                                                        80       90
ASZ85160.1_phosphoprotein_Rhabdoviridae   .....KVDWDQEEFKLDL.TSCSR.PLIQDFMK................................
sp|Q9E7N7|_Phosphoprotein_Lettuce_necro   AVKD.........L.LEL.AAKSQGIVVTDVMQ................................
sp|P06747|PHOSP_RABVP_Phosphoprotein      ..AKVGEGKYREDFQMDE.GEDPS.LLFQSYLD................................
sp|Q9E787|PHOSP_BEFVB_Phosphoprotein      TPKL.........S.WNDEFENCT.........................................
sp|Q82681|PHOSP_IHNVW_Phosphoprotein      QLIL.........K.YVE.EESC..........................................
sp|Q6E0X0|PHOSP_MMVR_Phosphoprotein_OS_   ...E.........R.HAV.MAPTQ.PSLSDVIN................................
sp|P03520|PHOSP_VSIVA_Phosphoprotein_OS   ..PE.........A.EQV.EGFIQ.GPLDDYADEEVDVVFTSDWKPPELESDEHGKTLRLTSPEG
gi|1070619313|ref|YP_009301740.1|_putat   PEED.........M.IEL.VLPY..........................................
TbRV_1_unnamed_protein_product            SSRK.........T.IEE.GEDQQ.........................................

                                              100                 110                     120
ASZ85160.1_phosphoprotein_Rhabdoviridae   ...ETIVQLVSW..........INSKVPL...............GCGTFYCDSDK.EGE......
sp|Q9E7N7|_Phosphoprotein_Lettuce_necro   ...NTAIALHHNLGLDASSLDWFVAGITF...............ANNSMI...ME.KMV.SAIKE
sp|P06747|PHOSP_RABVP_Phosphoprotein      ...NVGVQIVRQ..........IRSGERF...............L..KIWS..QTVEEI......
sp|Q9E787|PHOSP_BEFVB_Phosphoprotein      .....YKGYVVS..........SEDLCNDSGNQEKNEVPIKQS......................
sp|Q82681|PHOSP_IHNVW_Phosphoprotein      ......QDALKD..........FGGL..IANIRQAHQAEMTSH......................
sp|Q6E0X0|PHOSP_MMVR_Phosphoprotein_OS_   ...EERQ.................APITF...............GNPPEV...MANARL.SALGY
sp|P03520|PHOSP_VSIVA_Phosphoprotein_OS   LSGEQKSQWLST..........IKAVVQS.............AKYWNLAECTFEA.SGEGVIMKE
gi|1070619313|ref|YP_009301740.1|_putat   ....MEIDLARK.........V.TEAIDL...............ALKDLI...IDR.SL.PRIKY
TbRV_1_unnamed_protein_product            .....GAKIAKD..........MEKKLYLTKELSHLNPSVRTKLEFDMSKIKEEV.HTEVILMIQ

                                                                  130       140                 150
ASZ85160.1_phosphoprotein_Rhabdoviridae   ......................IRLIHKNYPNNSQLL....EELKE....LE....KS.......
sp|Q9E7N7|_Phosphoprotein_Lettuce_necro   LQIEVRNIQVASSGIK.G..TSEELVSKMKANKNDIV....KELVK....TR....DSVLSAMGG
sp|P06747|PHOSP_RABVP_Phosphoprotein      ......................ISYVAVNFPNPPGKS....SEDKS....TQ....TT.....GR
sp|Q9E787|PHOSP_BEFVB_Phosphoprotein      ...................................SLE....DVAQ....VL....SL.......
sp|Q82681|PHOSP_IHNVW_Phosphoprotein      ...................................LEK....VATE....HR.............
sp|Q6E0X0|PHOSP_MMVR_Phosphoprotein_OS_   DNLTEREKRILAVGVRCG..EAAKDYHSLTTTKKWIEDELKSQMVALASSTRTLTEAASLHTTFA
sp|P03520|PHOSP_VSIVA_Phosphoprotein_OS   RQIT.............PDVYKVTPVMNTHPSQSEAV....SDVWS....LS....KT.......
gi|1070619313|ref|YP_009301740.1|_putat   MMVPKPKEA.....................................................T..
TbRV_1_unnamed_protein_product            S..................................TLQ....PYSQ....LF....DL...D.FK

                                                  160                 170                 180
ASZ85160.1_phosphoprotein_Rhabdoviridae   ..LVETPAPSAPIR.EIDV.......RDR...KII.....IL.SR..SDDPP.............
sp|Q9E7N7|_Phosphoprotein_Lettuce_necro   IL....SAPEIEQQPVKTVTIGASQGRRK...STVVPPIEIN.PE..LESPVL..SKTVSTA.TP
sp|P06747|PHOSP_RABVP_Phosphoprotein      ELKK.ETTPTPSQR.ESQS.......SKA...RMA.....AQ.TA..SGPPALEWS....AT.NE
sp|Q9E787|PHOSP_BEFVB_Phosphoprotein      ..FQIRSEVDYKIE.KDNK.......NQVKIIKLS.....KQDKSVKSKRNDV........V.NQ
sp|Q82681|PHOSP_IHNVW_Phosphoprotein      ..........ANLQ.ALTK.......SQQEHEKVS.....KEILSAVIAIRSN........LNEN
sp|Q6E0X0|PHOSP_MMVR_Phosphoprotein_OS_   ML....HSPSIKRKAE.................AM.SH..IS.QG..EESIDI..SKLNKTG.ME
sp|P03520|PHOSP_VSIVA_Phosphoprotein_OS   ..SMTFQPKKAS.....................................................
gi|1070619313|ref|YP_009301740.1|_putat   PP....PPPLQP...PQET.......STP...PMQ.....AQ.PS..TSMPKR..S...GPL.PP
TbRV_1_unnamed_protein_product            V.NAPSATPETNIR.EQTI.......N.....HTA........PSIETKAPEL..T....TL.QP
```

```
                                                                                         190
                                                                                           .
ASZ85160.1_phosphoprotein_Rhabdoviridae   ...................WTG.TLVQLAGSREKAHNP...........................
sp|Q9E7N7|_Phosphoprotein_Lettuce_necro   ...EERIRH..EKEKLLAD..LDWEIGEI...AQYTPL......IV..DFLVP..DDIL......
sp|P06747|PHOSP_RABVP_Phosphoprotein      ...EDDLSV..EAEIA.HQIAE.SFSKKYKFPSRSSGILLYNFEQL..KMNL...DDIVKEAKNV
sp|Q9E787|PHOSP_BEFVB_Phosphoprotein      ...DSDKHMKYDSNFDAVM..D.QFQKGIRIKKRFGKGY.VKINA.DNMPGT.Y.HDLSNVIS..
sp|Q82681|PHOSP_IHNVW_Phosphoprotein      HSPLP.KPLDPDQVKAARA..L.GFGIGYRTALNVFDR..IKGVTPDNAGSQ.EVKNL..AIR..
sp|Q6E0X0|PHOSP_MMVR_Phosphoprotein_OS_   ...DIWVAM..ESESKEDA..VDTYLRNI...LEVDPTQ..FYAID..GWGLY..LDFIPTWH..
sp|P03520|PHOSP_VSIVA_Phosphoprotein_OS   ..................L..Q.PLTISLD.ELFSSRGEF..................I......
gi|1070619313|ref|YP_009301740.1|_putat   ...TESTSR..ERERSALV..V.EFMKGVKLVGIYEPEQTYTLKIG..VGGVT..EDLI......
TbRV_1_unnamed_protein_product            ...TNVTTK...LQ.KEWL..T.KLKKGIKCKSRSEENEYYSFKID..NKNIKE.SDVSNCLQ..


                                        200                            210             220         230                        240
                                          .                              .               .           .                          .
ASZ85160.1_phosphoprotein_Rhabdoviridae   DFN........STTPVVDMVIW.ALMQGKQYNRFALRYQIEEAEF.....DEED......D
sp|Q9E7N7|_Phosphoprotein_Lettuce_necro   ...A.M.AADGLTPEL..............KEKIQNEIIENHIAL.MAL.EE......YS.S
sp|P06747|PHOSP_RABVP_Phosphoprotein      PGVTRL.ARDGSKLPLRCVLGWVALAN...SKK..FQLLVESNKLSKIM.QDDLNR..YTSC
sp|Q9E787|PHOSP_BEFVB_Phosphoprotein      TV........KGEKT...............VEEMIRYLFKKSKRFKSIN.KTLNIDEMIL.C
sp|Q82681|PHOSP_IHNVW_Phosphoprotein      AA.EED.EYEGSPTF...............FRKVIDAVKKRMK...........QG.....Q
sp|Q6E0X0|PHOSP_MMVR_Phosphoprotein_OS_   ...Y.I.AAGKNSAQF..............KTSYADEIVEQRAAFERVLSKR.PR...VE.I
sp|P03520|PHOSP_VSIVA_Phosphoprotein_OS   .....S.VGGDGRMSHKE..............AILLGLR.YKKLYNQAR.VK......YS.L
gi|1070619313|ref|YP_009301740.1|_putat   ...N.EVNEQGLDQP...............TQEFIENLLCRLEYGQTLH.SL......YELA
TbRV_1_unnamed_protein_product            IL.......TLNN.................KEEVMFNILSLHPQVRQLA.AMFVLP..LN.L
```
