# Supplementary material for: The First Complete Genome Sequence of a Novel Tetrastichus brontispae RNA Virus-1 (TbRV-1)
Source: Viruses. 2019 Mar 13;11(3):257. doi: 10.3390/v11030257 (PMC6466307; doi:10.3390/v11030257)
Supplement: Supplementary file 1 [file viruses-11-00257-s001.zip › supplementary files/Supplementary Table S2.docx]

Supplementary Table S2 Sequences used for phylogenetic tree construction in this study

|  | Accession number | Virus species | Family | genera | **Virus abbreviation** |  |
| --- | --- | --- | --- | --- | --- | --- |
| 1 | YP_425092.1 | Lettuce necrotic yellows virus | Rhabdoviridae | Cytorhabdovirus | LYNV | Reference strain |
| 2 | NP_597914.1 | Northern cereal mosaic cytorhabdovirus |  |  | NCMCV | / |
| 3 | YP_006576506.2 | Persimmon virus A |  |  | PVA | / |
| 4 | APG78723.1 | Hubei dimarhabdovirus 2 |  | Dimarhabdovirus | HuDV2 | / |
| 5 | AJG39196.1 | Wuhan insect virus 7 |  |  | WuIV7 | / |
| 6 | AJG39212.1 | Wuhan Louse Fly virus 10 |  |  | WuLF10 | / |
| 7 | APG78684.1 | Hubei lepidoptera virus 2 |  |  | HuLV2 | / |
| 8 | NP_065409.1 | Bovine ephemeral fever virus |  | Ephemerovirus | BEFV | Reference strain |
| 9 | YP_003518294.1 | Ngainganhapavirus |  | Hapavirus | NHV | / |
| 10 | YP_002333280.1 | Wongabelhapavirus |  |  | WHV | / |
| 11 | AHB08865.1 | Kolente virus |  | Ledantevirus | KolV | / |
| 12 | BAN29060.1 | Nishimuroledantevirus |  |  | NLV | / |
| 13 | NP_056797.1 | Rabies lyssavirus |  | Lyssavirus | RABV | Reference strain |
| 14 | NP_919035.1 | Hiramenovirhabdovirus |  | Novirhabdovirus | HNRV | / |
| 15 | NP_042681.1 | Infectious hematopoietic necrosis virus |  |  | IHNV | Reference strain |
| 16 | YP_052855.1 | Maize mosaic nucleorhabdovirus |  | Nucleorhabdovirus | MMNV | Reference strain |
| 17 | YP_003126913.1 | *Drosophila melanogaster* sigmavirus AP30 |  | Sigmavirus | DmSV | / |
| 18 | YP_008686601.1 | *Drosophila obscura* sigmavirus 2 |  |  | DoSV | / |
| 19 | YP_002308576.1 | Lettuce big vein associated varicose virus |  | Vesiculovirus | LBVAVV | / |
| 20 | NP_041716.1 | Vesicular stomatitis Indiana virus |  |  | VSIV | Reference strain |
| 21 | YP_009094323.1 | *Culex tritaeniorhynchus* rhabdovirus |  | Unclassified | CtRV | / |
| 22 | AHL66985.1 | Long Island tick rhabdovirus |  |  | LITRV | / |
| 23 | AJG39218.1 | Wuhan Mosquito virus 9 |  |  | WuMV9 | / |
| 24 | YP_009259650.1 | *Diachasminorpha longicaudata* rhabdovirus |  |  | DlRHV | / |
| 25 | NP_071471.1 | Newcastle disease virus B1 | Paramyxoviridae | Avulavirus | NDVB1 | Reference strain |
| 26 | NP_056924.1 | Measles morbillivirus |  | Morbillivirus | MMV | Reference strain |
| 27 | NP_604442.1 | Human respirovirus 1 |  | Rubulavirus | HRSV1 | Reference strain |
| 28 | sp\|O55528 | Sendai virus |  |  | SdV | Reference strain |
| 29 | NP_054714.1 | Mumps rubulavirus 2 |  |  | MRV2 | Reference strain |
| 30 | YP_012613.1\| | Human metapneumovirus | Pneumoviridae | Metapneumovirus | HMV | Reference strain |
| 31 | NP_042024.3 | Borna disease virus 1 | Bornaviridae | Bornavirus | BDV1 | Reference strain |
| 32 | NP_066251.1 | Zaire ebolavirus | Filoviridae | Ebolavirus | ZEV | Reference strain |
| 33 | AHW76811.1 | *Sclerotinia sclerotiorum* negative-stranded RNA virus 1 | Mymonaviridae | Sclerotimonavirus | SsNSRV1 | Reference strain |
| 34 | YP_002905331.1 | Midway nyavirus | Nyamiviridae | Nyavirus | MNV | Reference stain |
| 35 | YP_009094051.1 | Sunshine coast virus | Sunviridae | Sunshinevirus | SShV | Reference strain |
| 36 | YP_009302387.1 | Xincheng Mosquito Virus 2 | unassigned Mononegavirales | / | XMV 2 | / |
| 37 | AJG39227.1 | Xincheng Mosquito Virus |  | / | XMV | / |
| 38 | YP_009304558.1 | Wenzhou Crab virus 1 |  | / | WzhCV | / |
| 39 | YP_009303699 | Lishi spider vius |  | / | LSV2 | / |
| 40 | Target | *Tetrastichus brontispae* RNA virus 1 |  | / | TbRV-1 | / |
