# Supplementary material for: The First Complete Genome Sequence of a Novel Tetrastichus brontispae RNA Virus-1 (TbRV-1)
Source: Viruses. 2019 Mar 13;11(3):257. doi: 10.3390/v11030257 (PMC6466307; doi:10.3390/v11030257)
Supplement: Supplementary file 1 [file viruses-11-00257-s001.zip › supplementary files/Supplementary Table S1.docx]

Supplementary Table S1 Primers used in this study

| **Primer** | **Oligonucleotide (5'→3')** | **Position (5’)** | **Application** |
| --- | --- | --- | --- |
| Dima1F | ATGGACCAGCTTGGTATGATTA | 17563 F | To assemble viral genome |
| Dima1R | TTGGGTTTAGTAGAGGGTGTTT | 19568 R |  |
| Dima2F | GTAGCACATCTAAACACTTTCCAACAC | 23051F |  |
| Dima2R | AAAAAGGTTCCCAACAGTGCTC | 14453R |  |
| Dima3F | ACTAACCTTCCAAACATACACC | 19568F |  |
| Dima3R | GGTCCGAAAGTACAGTTATTAA | 14453R |  |
| Dima 3GSP | AGATTGTGTCTGCTTGGTATCCGGGTTC | 14453 | Determination of genome termini |
| Dima 5GSP | GTCACTGCTTTACCAGTGGTTCTTCG | 132 |  |
| VS-1 | CTTAAAAACCGCTAAAGAGGTA | 1 | CDS validation |
| VA-1 | TTGAGAACTATTTGAAGACAGG | 1562 |  |
| VS-2 | CAAGATTATATCACAACCTGTC | 1525 |  |
| VA-2 | GGACTCTGAATTAGTTCCTATT | 2608 |  |
| VS-3 | GAACTAATTCAGAGTCCTTGAA | 2592 |  |
| VA-3 | ATACCCGATAGAAAGAATAAAG | 3629 |  |
| VS-4 | TTCTTAATAACTGTACTTTCGGACC | 3605 |  |
| VA-4 | ATAAGGAACAAATCCTCAGCATC | 5711 |  |
| VS-5 | TGCAGAGAGTGACTATGCTGAAA | 5660 |  |
| VA-5 | GTTGCTTTGTCTAACACAGATGAAT | 9500 |  |
| VS-6 | CAAATTAAGAGATGCAATCCATTGG | 9300 |  |
| VA-6 | AACGTAGTCCCGAAATTGTAGGG | 10694 |  |
| VS-7 | TAGAGATCTTGGTGCAGTTGGTT | 10195 |  |
| VA-7 | TTCAAGTCTATGGATGTAGTCGT | 12170 |  |
| QVA-1 | AGTCCAATAAGTGCTAAAACTGCAA | RdRP | Quantification of TbRV-1 |
| QVS-1 | ACTCACTTAGGAACCTTGGAAACA | RdRP |  |
| MS | ATGAATCCTAAACACCCTCTACTAAAC | Matrix | Validation the presence of virus |
| MA | CCCTGGTCTTTGACTAGGAGTC | Matrix |  |
